# A guide to writing case reports for the *Journal of Medical Case Reports* and *BioMed Central Research Notes*

**DOI:** 10.1186/1752-1947-7-239

**Published:** 2013-11-27

**Authors:** Richard A Rison

**Affiliations:** 1Presbyterian Intercommunity Hospital Health Stroke Program, Los Angeles County Medical Center, University of Southern California Keck School of Medicine, 12401 Washington Blvd, Whittier, CA 90602, USA

## Abstract

Case reports are a time-honored, important, integral, and accepted part of the medical literature. Both the *Journal of Medical Case Reports* and the Case Report section of *BioMed Central Research Notes* are committed to case report publication, and each have different criteria. *Journal of Medical Case Reports* was the world’s first international, PubMed-listed medical journal devoted to publishing case reports from all clinical disciplines and was launched in 2007. The Case Report section of *BioMed Central Research Notes* was created and began publishing case reports in 2012. Between the two of them, thousands of peer-reviewed case reports have now been published with a worldwide audience. Authors now also have Cases Database, a continually updated, freely accessible database of thousands of medical case reports from multiple publishers. This informal editorial outlines the process and mechanics of how and when to write a case report, and provides a brief look into the editorial process behind each of these complementary journals along with the author’s anecdotes in the hope of inspiring all authors (both novice and experienced) to write and continue writing case reports of all specialties. Useful hyperlinks are embedded throughout for easy and quick reference to style guidelines for both journals.

## Introduction: the importance of case reports

Case reports are a time-honored tradition in the medical profession. From Hippocrates (460 B.C. to 370 B.C.), and even arguably further back since the papyrus records of ancient Egyptian medicine (c. 1600 B.C.) to modern day, physicians of all specialties have described interesting cases involving all specialties [1,2]. Published case reports provide essential information for optimal patient care because they can describe important scientific observations that are missed or undetected in clinical trials, and provide individual clinical insights thus expanding our knowledge base [3].

The publication of case reports has indeed become a standard lexicon of the medical literature. Examples abound. Few practicing physicians would not know for instance the significance and subsequent discovery of a disease whose first description in 1981 began with the title in the medical case report literature as: “A preliminary communication on extensively disseminated Kaposi’s sarcoma in a young homosexual man” [4]. There is no neurologist that I know who is unfamiliar with the disease whose description began in 1817 by James Parkinson (1755 to 1824) with the title “An essay on the shaking palsy.” [5].

Yes, both of the above-mentioned famous diseases (the acquired immunodeficiency syndrome and Parkinson’s disease) were first described in the case study format. The act of recording, discussion with colleagues, and publishing our clinical observations with patients remains essential to the art of medicine and patient care. As Osler once said “Always note and record the unusual…Publish it. Place it on permanent record as a short, concise note. Such communications are always of value.” [6].

But how and when should we do this? Early case reports were little more than personal communications between colleagues about unique and interesting patients seen in their respective medical practices. This anecdotal reporting has evolved into an accepted form of scholarly publication with the ability to rapidly disseminate knowledge to a broad medical audience [7] using the generally accepted format of a title, abstract, introduction (background), case presentation, discussion, conclusions, and references. Many biomedical journals publish case reports and provide authors with guidelines that provide instruction for acceptance criteria, content, and format and give advice on relevant patient case reports that merit publication [3].

There are already many well-written published articles on how and when to write a good case report (please see Recommended further reading section at the end). I will not re-invent the wheel, but within this editorial I hope to provide an informal guide on how and when to write a case report for BioMed Central (BMC), in particular the *Journal of Medical Case Reports* (*JMCR*) and *BioMed Central Research Notes* (*BMCRN*). The utility of the newly created Cases Database will also be discussed. Relevant and useful website links will be used throughout to allow the reader easy access to further information on BMC requirements. I also hope to impart to the reader a brief overview of case report editorial flow in both *JMCR* and *BMCRN* along with the complementary relationship between both journals. I will also give anecdotes of how I personally approach things.

## Definitions

What exactly is a case report? From peer-reviewed journals to Wikipedia (and yes, I read Wikipedia like we all do) definitions are readily available and generally agreed upon. A simple online search shows the following definition from “thefreedictionary.com” [8]: “Case Report A report of a single case of a disease, usually with an unexpected presentation, which typically describes the findings, clinical course, and prognosis of the case, often accompanied by a review of other cases previously reported in the biomedical literature to put the reported case in context.” Wikipedia [9] has this to say: “In medicine, a case report is a detailed report of the symptoms, signs, diagnosis, treatment, and follow-up of an individual patient. Case reports may contain a demographic profile of the patient, but usually describe an unusual or novel occurrence. Some case reports also contain a literature review of other reported cases.” Whether one uses the above definitional references or older more classic ones [10], all are in agreement.

## How to start: the patient

Things start at the bedside or in the office with the most important person involved: the patient. Patients and their stories (including from their friends, coworkers, and family) are our portal to writing the case report. Patients (both in-patients and out-patients) are assessed, we confer with colleagues, appropriate investigations then follow, and treatment if possible begins. If I encounter an in-patient on call then I follow him or her throughout his or her hospitalization and, I hope, timely discharge. The patient is then followed and reexamined in the office over the course of time to see how the clinical course evolves. I usually wait 6 months over the course of multiple visits before I actually begin to write a case report so as to allow enough time for the clinical course to play out. Of course if the patient is hospitalized with an acute and rapid illness then this time may be much shorter, but I still follow him or her with daily neurologic examinations.

## Collegial discussion and the Internet: our modern day water cooler

When an interesting condition is encountered in either the hospital or the office setting, I discuss the case in person with both my local neurology colleagues and colleagues of other specialties to see if they have encountered before the clinical scenario that I am dealing with at the time. This is usually a quick face-to-face nursing station conversation. If the case is particularly challenging then I will contact my local university colleagues for their opinion (especially if an urgent transfer needs to be arranged). I then “hit the books”, or at least I used to. Nowadays I usually “hit the keyboards” which are plentiful at every hospital nursing station and in my office. Indeed, the Internet seems to have become our modern day replacement for office water cooler conversations. Since it is readily available (and free to me because I am a member of the staff) in the hospital in which I see patients and in my office, I usually start with UpToDate® [11] and then click the links to individual references. Further reading is then supplemented by both PubMed [12] (free) and Cases Database (also free) [13] (see later). If I feel that a particular patient warrants a case report, then I continue to read more and more. There are also medical list servers and medical online communities to which one can post a case with de-identified images online and petition the advice of colleagues worldwide. I use both Neurolist [14] (a membership-only service, but membership is free) and The American Academy of Neurology (AAN) for my specialty and/or subspecialties [15] (also a membership-only service, the fee of which comes out of my yearly AAN dues). Another useful list server is sermo® [16], which has free membership. Teaching grand rounds at one’s local university or hospital, poster presentations, and simple discussion with professors giving lectures at local seminars are also good (and previously “traditional”) places to start. I have always preferred an in-person encounter to discuss a case with a colleague or professor, but given the current day and age (daily workload, travel costs, time away from the office and family, and so on), I have found Internet-based discussion (keeping all patient information anonymous of course) very helpful.

## The BMC series, *JMCR*, and *BMCRN*: a brief history

The BMC series is a group of open access, peer-reviewed journals that spans most areas of biological and clinical research. There are currently 65 journals in the series, including (alphabetically) BMC Anesthesiology to BMC Women’s Health. Some of these publish case reports within their respective disciplines, and some do not [17].

*JMCR* is an online, open access journal under BMC auspices dedicated mainly to the publication of high quality case reports, and aims to contribute to the expansion of current medical knowledge (please see specific publication criteria below). It was created and founded by Michael Kidd and colleagues in 2007 and at the time was believed to be the world’s first international medical journal devoted to publishing case reports from all clinical disciplines. In the 5 years since its launch, *JMCR* has published over 2000 case reports. In 2011, case reports were downloaded from the journal’s website over 1,500,000 times [18].

*BMCRN* is also an online, open access journal under BMC auspices publishing scientifically sound research across all fields of biology and medicine. The journal provides a home for short publications, case series, and incremental updates to previous work with the intention of reducing the loss suffered by the research community when such results remain unpublished. *BMCRN* began publishing case reports in 2012 and now has a dedicated section for case reports [19].

Please read on to see the complementary relationship of case reporting between the two journals, how they relate to other journals in the BMC series, and further information on editorial work flow including specific publication criteria.

## Cases Database: an invaluable resource

Since the launch of *JMCR* in 2007 and the more recent introduction of case reports to the *BMCRN*, which aims to have a broader scope, BMC has acknowledged and continues to acknowledge the value of case reports to the scientific literature. To further strengthen this commitment, BMC in conjunction with Michael Kidd have developed the invaluable new resource of Cases Database, a continually updated, freely accessible database of thousands of medical case reports from multiple other publishers, including Springer, British Medical Journal, and PubMed Central. By aggregating case reports and facilitating comparison, Cases Database provides a simple resource to clinicians, researchers, regulators and patients to explore content and identify emerging trends [20].

http://www.casesdatabase.com/

I find Cases Database indispensable when I research a particular patient’s condition. It is very helpful in seeing if a particular condition has been reported before and what treatment the authors have performed. It is an invaluable resource which can be used to check and see if previous cases have been reported before and how other authors have managed their patients with similar clinical conditions. When I last checked, Cases Database had in its repository 27,915 peer-reviewed medical case reports from 250 journals (!) [13]. Cases Database is quickly becoming my first go to when reading about a patient’s condition and symptoms.

## When to write a case report

How does one determine when to write an actual case report? What constitutes and what are the criteria for publication? Different journals have different criteria, but here are the criteria for *JMCR* and *BMCRN*.

*JMCR*[21] publishes original and interesting case reports that contribute significantly to medical knowledge. Manuscripts must meet one of the following criteria: unreported or unusual side effects or adverse interactions involving medications; unexpected or unusual presentations of a disease; new associations or variations in disease processes; presentations, diagnoses and/or management of new and emerging diseases; an unexpected association between diseases or symptoms; an unexpected event in the course of observing or treating a patient; findings that shed new light on the possible pathogenesis of a disease or an adverse effect.

http://www.jmedicalcasereports.com/authors/instructions/casereport

*BMCRN*[22] has somewhat different publication criteria: *BMCRN* considers medical case reports that describe any clinical case. Case reports submitted to *BMCRN* do not need to be novel, but must be authentic cases and have some educational value along with representing at least an incremental advance in the field. *BMCRN* will not consider case reports describing preventive or therapeutic interventions because these generally require stronger evidence.

http://www.biomedcentral.com/bmcresnotes/authors/instructions/casereport

Neither *BMCRN* nor *JMCR* will consider case reports where there are ethical concerns.

*JMCR* and *BMCRN* have the following definitions that authors should know: a single case report, two case reports, or a case series (greater than two reported cases). Both journals follow this format and accept submissions with these title structures.

I tend to classify case reports in my mind generally as follows: diagnosis-related, management-related, or both [10]. Either type should have clear and concise take-home messages and teaching points. I personally keep a stack of charts labeled “Curious Cases” on a bookshelf within my small office next to my desk which is always within my field of view at work, adhering to the “out of sight, out of mind” principle. Over the years that space has grown and, admittedly, I have cases dating back over the entire span of my years in practice (now over 13 years) which I simply have not gotten around to yet (!).

## BMC editorial workflow for case reports: a brief glimpse

If a BMC Series journal editorial team considers a submitted case report unsuitable for their respective specialty journal (and now a growing list of Springer journals that BMC is now affiliated with), the authors are given the option to transfer their manuscript to *BMCRN*. If this option is exercised, then the BMC editorial team (usually the Case Report Section Editor for *BMCRN* in conjunction with the appropriate Associate Editor) determines if the manuscript is suitable for BMCRN or if it is more suitable for *JMCR* (based on the criteria listed above). The manuscripts will then be forwarded on to the respective Deputy and/or Associate Editors for peer review depending on which of the journals the author(s) agree(s) to. Peer reviewers are solicited (usually at least one at *BMCRN* and at least two at *JMCR*). The peer review comments (which are open and identifiable at *JMCR* and blinded at *BMCRN*) are then usually sent to the authors for appropriate revisions and rebuttals (unless it is felt that the manuscript should be rejected outright, at which time the editorial office sends the authors an explanatory letter). After these revisions and rebuttals have been performed, the revised manuscript and rebuttals are sent back to the respective editors for a final decision and recommendations. These decisions and recommendations are then forwarded on to the Editor-in-Chief for final approval for publication. At *JMCR*, manuscripts are professionally copyedited before being sent off to the production team for publication, whereas at *BMCRN* the authors are requested to obtain their own professional copyediting (if needed) before publication (the respective costs being reflected within the different article processing charges for both journals). When the manuscripts are published in both journals, they are in the preliminary form before being converted to the final form after production.

Author satisfaction consistently ranks high for the overall process in both journals.

## The actual case report

Now let us discuss the brass tacks of writing the actual case report by going through the individual sections that will comprise the manuscript. I will present them in a sequence that matches the journals’ website requirements and provide easily accessible hyperlinks to both respective journals.

## Title page

The first page of the manuscript should be a dedicated title page, including the title of the article. The title should be a clear and short description of the case with a list of the full names, institutional addresses and email addresses for all authors. There should always be at least one corresponding author who is clearly identified. Abbreviations within the title should always be avoided.

http://www.jmedicalcasereports.com/authors/instructions/casereport#formatting-title

http://www.biomedcentral.com/bmcresnotes/authors/instructions/casereport#title

I usually end the title with “…: a case report” or “…: two case reports” or “…: a case series”. I also try to avoid any puns or overly cute wording within the title and try to keep things strictly descriptive and clear. The title needs to accurately describe the case – after all, this may be all that someone reads. If a cute or clever title is used that obscures what the case is really about, then it may be even less likely that the manuscript is read.

## Abstract

The Abstract should be “short and sweet”. It should not exceed 350 words. Abbreviations or references within the Abstract should not be used. The Abstract should be structured into three sections: Background, an introduction about why this case is important and needs to be reported. Please include information on whether this is the first report of this kind in the literature; Case presentation, brief details of what the patient(s) presented with, including the patient’s age, sex and ethnic background; Conclusions, a brief conclusion of what the reader should learn from the case report and what the clinical impact will be. Is it an original case report of interest to a particular clinical specialty of medicine or will it have a broader clinical impact across medicine? Are any teaching points identified?

http://www.jmedicalcasereports.com/authors/instructions/casereport#formatting-abstract

http://www.biomedcentral.com/bmcresnotes/authors/instructions/casereport#abstract

I find this is the most important part because this is often all that people will read and its availability will allow easy retrieval from electronic databases and help researchers decide their level of interest in the case report. The Abstract should be a concise and condensed version of the case report and should include the same main sections of the main text and be as succinct as possible [3]. This is the last thing that I usually write as it tends to flow easily after I have invested my time in thought and writing of the manuscript.

## Keywords

This section is comprised of three to ten keywords representing the main content of the article. It is important for indexing the manuscript and easy online retrieval.

http://www.jmedicalcasereports.com/authors/instructions/casereport#formatting-keywords

http://www.biomedcentral.com/bmcresnotes/authors/instructions/casereport#formatting-keywords

## Introduction (Background)

The Introduction (*JMCR*) or Background (*BMCRN*) section should explain the background of the case, including the disorder, usual presentation and progression, and an explanation of the presentation if it is a new disease. If it is a case discussing an adverse drug interaction the Introduction should give details of the drug’s common use and any previously reported side effects. It should also include a brief literature review. This should give an introduction to the case report from the standpoint of those without specialist knowledge in the area, clearly explaining the background of the topic. It should end with a very brief statement of what is being reported in the article.

http://www.jmedicalcasereports.com/authors/instructions/casereport#formatting-intro

http://www.biomedcentral.com/bmcresnotes/authors/instructions/casereport#background

The Introduction or Background serves as the sales pitch for the rest of the manuscript. It should be concise and salient [3] and immediately attract the reader’s attention to entice him or her to read on.

## Case presentation

This should present all relevant details concerning the case. The Case presentation section should contain a description of the patient’s relevant demographic information (without adding any details that could lead to the identification of the patient); any relevant medical history of the patient; the patient's symptoms and signs; any tests that were carried out and a description of any treatment or intervention. If it is a case series, then details must be included for all patients. This section may be broken into subsections with appropriate subheadings.

http://www.jmedicalcasereports.com/authors/instructions/casereport#formatting-case

http://www.biomedcentral.com/bmcresnotes/authors/instructions/casereport#presentation

This is one of the most integral sections. The case should be described in a concise and chronological order. One should usually begin with the primary complaint, salient history (including significant family, occupational, and other social history along with any significant medications taken or allergies), followed by the physical examination, starting with the vital signs presented at the examination, along with pertinent investigations and results. There should be enough detail (but not too much) for the reader to establish his or her own conclusions about the validity. It should contain only pertinent information and nothing superfluous or confusing [3].

## Discussion

This is an optional section in *JMCR* for additional comments that provide additional relevant information not included in the case presentation, and that put the case in context or that explain specific treatment decisions.

http://www.jmedicalcasereports.com/authors/instructions/casereport#formatting-discussion

This section should evaluate the patient case for accuracy, validity, and uniqueness and compare and contrast the case report with the published literature. The authors should briefly summarize the published literature with contemporary references [3].

Although this section is optional in *JMCR* (and not even listed separately on the *BMCRN* guidelines website), I find that most authors write this section, or an expanded conclusions section incorporating the elements listed above.

I personally write a separate discussion section and conclusions section for each case report that I author.

## Conclusions

This should state clearly the main conclusions of the case report and give a clear explanation of their importance and relevance. Is it an original case report of interest to a particular clinical specialty of medicine or will it have a broader clinical impact across medicine? Information should be included on how it will significantly advance our knowledge of a particular disease etiology or drug mechanism (if appropriate).

http://www.jmedicalcasereports.com/authors/instructions/casereport#formatting-conclusion

http://www.biomedcentral.com/bmcresnotes/authors/instructions/casereport#conclusions

This should be short and concise with clear take-home messages and teaching points [3].

## Patient’s perspective

This section is an opportunity for patients to add a description of a case from their own perspective. The patients should be encouraged to state what originally made them seek medical advice, give a description of their symptoms, whether the symptoms were better or worse at different times, how tests and treatments affected them, and how the problem is now. This section can be written as deemed appropriate by the patients, but should not include identifying information that is irrelevant to the case reported. As medicine becomes more person-centered, the voice of the individual patient becomes even more important, both to assist in clinical decision making, and for medical education.

http://www.jmedicalcasereports.com/authors/instructions/casereport#formatting-patients

This optional section is unique to *JMCR*, and I believe adds an important new dimension to the traditional case report. Most authors still do not yet take advantage of this, but I hope as time goes on and more and more open access case report manuscripts are published that this section will be routinely used, not just in *JMCR* but also in *BMCRN* and all other BMC clinical journals. I recall one manuscript in particular where the patient himself was requesting publication as soon as possible because of his terminal disease. He wanted his message out there and be available to all to read before he died.

## List of abbreviations

When abbreviations are used in the text they should be defined in the text at first use, and a list of abbreviations can be provided, which should precede the Competing interests and Authors’ contributions sections.

http://www.jmedicalcasereports.com/authors/instructions/casereport#formatting-abbreviations

http://www.biomedcentral.com/bmcresnotes/authors/instructions/casereport#formatting-abbreviations

Both *JMCR* and *BMCRN* publish case reports over a wide range of medical and surgical specialties, and it is important for the reader who may not be within that particular specialty to readily access a quick list of common technical abbreviations. Also, given the open access nature of both journals, please keep in mind that non-medical professionals may read the manuscript as well.

## Consent

This section is compulsory for BMC. It should provide a statement to confirm that the patient has given their informed consent for the case report to be published. The written consent should not routinely be sent in along with the manuscript submission (because of patient privacy issues), but the BMC editorial office may request copies of the consent documentation at any time. The following wording is recommended: “Written informed consent was obtained from the patient for publication of this case report and accompanying images. A copy of the written consent is available for review by the Editor-in-Chief of this journal.” If the individual described in the case report is a minor, or unable to provide consent, then consent must be sought from his or her parents or legal guardians. In these cases, the statement in the ‘Consent’ section of the manuscript should be amended accordingly. Please keep in mind that manuscripts will not be peer reviewed if a statement of patient consent is not present.

http://www.jmedicalcasereports.com/authors/instructions/casereport#formatting-consent

http://www.biomedcentral.com/bmcresnotes/authors/instructions/casereport#consent

In practice, I always start with written consent from the patient. If the patient is incapacitated or deceased, then I obtain consent from the patient’s next-of-kin. Once this is obtained then I place it in the patient’s chart for safe keeping. I find that most patients and family members are quite agreeable to publication as long as their details are anonymous. BMC has very clear and explicit consent criteria and consent forms in multiple languages. I always keep a consent form within my office (and carry a few in my doctor’s handbag for hospital consults) for ready access. After I have obtained consent, I place it in the patient’s chart and keep it my office.

If the patient has died, then I try to obtain consent from the patient’s next-of-kin. This is usually done via telephone or postal mail. If the deceased patient’s family is amenable (and usually they are), then I send them (I never use email when it comes to patient-identifying information) the pre-filled out consent form in their language with a return envelope and paid for postage via the postal service. If I am unable to obtain consent this way in a case involving a patient who has died, then I write in the Consent section the following: “Written informed consent could not be obtained from the deceased patient’s next-of-kin for publication of this case report and accompanying images despite all reasonable attempts. Every effort has been made to protect the patient’s identity and there is no reason to believe that our patient would have objected to publication.”

If the patient was last known to be living but untraceable (or mentally incapacitated without next-of-kin consent), then I just simply do not publish the case.

For further information, please see *JMCR* and *BMCRN* website consent section hyperlinks as listed above.

## Competing interests

A competing interest exists when one’s interpretation of data or presentation of information may be influenced by a personal or financial relationship with other people or organizations. Authors must disclose any financial competing interests and should also reveal any non-financial competing interests that may cause embarrassment were they to become public after the publication of the manuscript. Authors are required to complete a declaration of competing interests. All competing interests that are declared will be listed at the end of published article. Where an author gives no competing interests, the listing should read “The author(s) declare that they have no competing interests”.

http://www.jmedicalcasereports.com/authors/instructions/casereport#formatting-competing

http://www.biomedcentral.com/bmcresnotes/authors/instructions/casereport#formatting-competing

I do not usually find any problems with competing interests in the case reports that I publish, but the section should always be completed in our era and in the spirit of complete disclosure.

## Authors’ contributions

In order to give appropriate credit to each author of a paper, the individual contributions of authors to the manuscript should be specified in this section.

An ‘author’ is generally considered to be someone who has made substantive intellectual contributions to a published study. To qualify as an author one should: 1) have made substantial contributions to conception and design, or acquisition of data, or analysis and interpretation of data; 2) have been involved in drafting the manuscript or revising it critically for important intellectual content; and 3) have given final approval of the version to be published. Each author should have participated sufficiently in the work to take public responsibility for appropriate portions of the content. Acquisition of funding, collection of data, or general supervision of the research group, alone, does not justify authorship. All contributors who do not meet the criteria for authorship should be listed in an Acknowledgements section. Examples of those who might be acknowledged include a person who provided purely technical help, writing assistance, or a department chair who provided only general support.

http://www.jmedicalcasereports.com/authors/instructions/casereport#formatting-contributions

http://www.biomedcentral.com/bmcresnotes/authors/instructions/casereport#formatting-contributions

I have found over the years a trend towards multi-authored case report manuscripts by many different individuals involved in the care of a patient(s). In my setting, it is usually me, a medical student or resident, a second-opinion tertiary colleague, and/or a pathologist or radiologist (if applicable). But I also recognize that there are situations that warrant more co-authors. The above criteria though for co-authorship should always be followed, and I have seen editorial situations where peer reviewers (including Associate Editors) have questioned what they felt was excessive authorship.

## Authors’ information

This section includes any relevant information about the author(s) that may aid the reader’s interpretation of the article and understanding of the standpoint of the author(s). This may include details about the authors’ qualifications, current positions they hold at institutions or societies, or any other relevant background information. Please refer to authors using their initials. Note this section should not be used to describe any competing interests.

http://www.jmedicalcasereports.com/authors/instructions/casereport#formatting-information

http://www.biomedcentral.com/bmcresnotes/authors/instructions/casereport#formatting-information

In practice, I have frankly also personally used this section to advertise my services and “tout” my certifications and subspecialties (along with any co-authors and affiliated institutions) to my surrounding local community. This has in turn given me a modest increase in business (which has been completely non-monetary to date), usually in the form of email-based queries, many of which come from patients outside of my locality.

## Acknowledgements

Authors should acknowledge anyone who contributed towards the article by making substantial contributions to conception, design, acquisition of data, or analysis and interpretation of data, or who was involved in drafting the manuscript or revising it critically for important intellectual content, but who does not meet the criteria for authorship. Also included should be the source(s) of funding for each author, and for the manuscript preparation. Authors must describe the role of the funding body, if any, in the: design, collection, analysis, and interpretation of data; writing of the manuscript; and decision to submit the manuscript for publication. Please also acknowledge anyone who contributed materials essential for the study. If a language editor has made significant revision of the manuscript, I recommend that you acknowledge the editor by name, where possible. Authors may also like to acknowledge (anonymously) the patient on whom the case report is based. If a scientific (medical) writer is used, this person should be included in the Acknowledgements section, including their source(s) of funding. Authors should obtain permission to acknowledge from all those mentioned in the Acknowledgements section.

http://www.jmedicalcasereports.com/authors/instructions/casereport#formatting-acknowledgements

http://www.biomedcentral.com/bmcresnotes/authors/instructions/casereport#formatting-acknowledgements

I have had colleagues who do not want to participate in the actual writing of the manuscript or do any actual “work” who have instead preferred to be mentioned in this section only.

## References

Authors must search for and cite published case reports that are relevant to the case they are presenting. There should be no more than 15 references usually, although BMC does publish manuscripts with more references particularly if there is an extended literature review. Unless it is of historic interest, please keep the references as contemporary as feasible (for example, within the last 5 years or so). Please avoid excessive referencing.

http://www.jmedicalcasereports.com/authors/instructions/casereport#formatting-references

http://www.biomedcentral.com/bmcresnotes/authors/instructions/casereport#formatting-references

## Cover letter

This is a separate document that should be written and uploaded with the main manuscript submission. I usually write this after I have written the Abstract. The cover letter should be addressed to the Editor-in-Chief in a formal manner and include all of the authors’ contact information. It should clearly and concisely state the title of the manuscript, and why the authors feel that their case report should be published based on any already available literature on the topic at hand. From an editor’s viewpoint, the cover letter is exceptionally important as that is the first thing that he or she reads and serves as the gateway to the Abstract and then the rest of the manuscript.

## BMC author academy: help for all

Both *JMCR* and *BMCRN* have a large number of non-native English-speaking authors. Since *JMCR* and *BMCRN* are both BMC publications whose editorial offices are based in England, the language of publication is of course English. The BMC author academy is a joint program by BMC and Edanz [23] aimed at equipping writers for successful publication. Their materials have been developed from training workshops that Edanz gives to researchers worldwide and are not just limited to case reports. BMC recommends Edanz for authors who want to have their manuscript edited by a native speaker of English who is a scientific expert. Edanz provides scientific editing and related services that raise the quality of manuscripts to the standard needed to be understood at peer review.

http://www.biomedcentral.com/authors/authoracademy

I find that most non-native English-speaking authors have their manuscripts reviewed informally by a native English-speaking colleague and/or friend who is usually mentioned within the Acknowledgements section. This is understandable to keep costs down. However, please be aware that poor grammar and frequent spelling mistakes can be an impediment to editorial work flow and peer review. The editorial staff for both *JMCR* and *BMCRN* are acutely aware and sensitive to this given the large number of international submissions. At both *JMCR* and *BMCRN*, submitted manuscripts with questionable grammar and spelling are returned back to the authors by the editorial staff if it is felt that the grammar and spelling mistakes would impede peer review. If these issues are minor and it is felt that they would not impede peer review, then the manuscripts are sent off to peer reviewers (when appropriate).

## Final checklist and the rule of **C**s

After I have completed a case report, I like to run through my long-winded (but useful) “rule of Cs” which is as follows.

Is it **
*C*
**lear, **
*C*
**oncise, and **
*C*
**oherent? Does it **
*C*
**onvey your message? Have you used **
*C*
**ases Database to look for any previously similar reported cases, and included them, if appropriate, in your references? Have you **
*C*
**onferred with your **
*C*
**olleagues on the **
*C*
**ontent? Will it **
*C*
**ause the reader to be **
*C*
**urious? Did you obtain **
*C*
**onsent? Does it **
*C*
**ontain all of the necessary information? Does it **
*C*
**omply with BM**
*C*
** guidelines? Do you think that it may need **
*C*
**opyediting? Do your **
*C*
**o-authors **
*C*
**oncur with the **
*C*
**ompleted paper? **
*C*
**an you **
*C*
**ut anything unnecessary out? Are your findings likely to be a **
*C*
**oincidence or by **
*C*
**hance alone? If so, then mention this in the Discussion section. Is the writing style **
*C*
**onsistent? Many times I find co-authored manuscripts have different writing styles within the same paper depending on who wrote what section. There should be a *C*entral, *C*orresponding author who is in *C*harge and oversees all of this. Is the **
*C*
**ase report written in a **
*C*
**hronological fashion with respect to the patient’s history and **
*C*
**hain of events? Is there anything that can be **
*C*
**ut out and have it still **
*C*
**ontain the **
*C*
**ompulsory information? Is it **
*C*
**oncise? Have you **
*C*
**onveyed **
*C*
**uriosity for your **
*C*
**ase report within your **
*C*
**over letter to the editorial team? Remember: your **
*C*
**over letter is the sales pitch to the editorial team! Make it **
*C*
**ount! Have you used within the manuscript **
*C*
**opyrighted information from another source? If so, do you need and/or have permission for use? After **
*C*
**ompletion, wait a **
*C*
**ouple of days before final submission to **
*C*
**lear your mind and read the manuscript again to **
*C*
**atch any mistakes that you may have made while you were **
*C*
**aught up in the **
*C*
**ompletion of it. Are the references **
*C*
**ontemporary? **
*C*
**an it be **
*C*
**omprehended by the average (“**
*C*
**”) reader? Remember, both *JMCR* and *BMCRN* are open access and freely available to anyone with an Internet **
*C*
**onnection and **
*C*
**omputer. **
*C*
**ast as wide a net as possible and **
*C*
**apture your **
*C*
**olleagues’ and other readers’ **
*C*
**uriosity. And first and foremost as a **
*C*
**linician: was the **
*C*
**are of your patient **
*C*
**ompetent and **
*C*
**ompassionate? (that is, are there any ethical concerns that may preclude peer review and publication?).

## Summary and parting advice

Case reporting can be fun and a lifelong hobby, both for novice and experienced authors alike. It is now integral and widely accepted within published medical literature and today’s electronic information and data-sharing age. By following the above recommended steps and general overview, I hope to encourage BMC authors to continue to write and submit manuscripts to both *JMCR* and *BMCRN*. After your manuscript is complete, please follow the rule of “Cs”, especially “**
*C*
**lear, **
*C*
**oncise, **
*C*
**oherent, **
*C*
**onsent, **
*C*
**ompassion, and **
*C*
**ompetence”, which will be appreciated by both reviewers and editors. Do not be afraid to obtain help from native English speakers for your manuscript. Also, please adhere to deadlines and follow instructions given by the editorial office, especially regarding any revisions. Editors read many different manuscripts and the longer it takes to get back a manuscript after revisions have been requested the less fresh that manuscript is in mind. Lastly, consider volunteering as an Associate Editor and/or reviewer within your specialty for both journals. I do for both, and the experience has improved both my writing and editing skills and daily interactions with patients.

## Recommended further reading

I recommend the following further instructive reading on how and when to write a case report: References [3,7,10,24] (the last referenced article is in German, but one should readily be able to obtain an English translation if needed through a local librarian. It is well worth reading.)

I also recommend the following instructive BMC-related editorials and commentaries concerning the modern-day importance of case reports: References 2, 18, and 19.
